# Opportunities and challenges of microbial siderophores in the medical field

**DOI:** 10.1007/s00253-023-12742-7

**Published:** 2023-09-27

**Authors:** Ajit Kumar Passari, Beatriz Ruiz-Villafán, Rodrigo Cruz-Bautista, Valerie Díaz-Domínguez, Romina Rodríguez-Sanoja, Sergio Sanchez

**Affiliations:** Instituto de Investigaciones Biomédicas, Universidad Nacional Autónoma de México, 04510 Mexico City, Mexico

**Keywords:** Siderophores, Antimicrobial, Antimalarial, Anticancer, Vaccines

## Abstract

Siderophores are low-molecular-weight secondary metabolites that function as iron chelators. Under iron-deficiency conditions, they are produced by a wide variety of microbes, allowing them to increase their iron uptake. The primary function of these compounds is the environmental iron scavenging and its transport into the cytosol. Iron is then reduced to its ferrous form to operate as an enzymatic cofactor for various functions, including respiration, nitrogen fixation, photosynthesis, methanogenesis, and amino acid synthesis. Depending on their functional group, siderophores are classified into hydroxamate, catecholate, phenolate, carboxylate, and mixed types. They have achieved great importance in recent years due to their medical applications as antimicrobial, antimalarial, or anticancer drugs, vaccines, and drug-delivery agents. This review integrates current advances in specific healthcare applications of microbial siderophores, delineating new opportunities and challenges as viable therapies to fight against diseases that represent crucial public health problems in the medical field.

**Key points**

• *Siderophores are low-molecular-weight secondary metabolites functioning as iron chelators*.

• *The siderophore’s properties offer viable options to face diverse clinical problems*.

• *Siderophores are alternatives for the enhancement of antibiotic activities*.

## Introduction

Iron is a necessary element in all living organisms; it is used as an enzyme cofactor to catalyze redox reactions associated with essential cellular functions such as respiration, DNA synthesis, energy production, and protection against reactive oxygen species (Di Fan and Qiaojun 2021; Seyoum et al. 2021).

Iron represents the fourth most abundant element in the Earth’s crust, although its bioavailability is significantly low (Osman et al. 2019). That occurs because iron exists predominantly in its oxidized ferric state (Fe^3+^), which is, to a great extent, insoluble at neutral pH levels. These conditions prevail in various familiar natural surroundings. In the characteristic habitat at neutral pH, the ferric iron has a solubility of 10^–17^ M. Neutral pH and aerobic conditions result in the oxidation of iron to an insoluble oxyhydroxide polymer (Osman et al. 2019). Siderophores are iron ion chelators of high affinity and specificity, with a molecular weight of less than 10 kDa. Under conditions of iron deficiency, they are released by various microbial systems, such as bacteria and fungi, and by individual plants. Several researchers reported siderophores production by Gram-positive and Gram-negative bacteria from different habitats under iron-deprived conditions (Abo-Zaid et al. 2020; Ghazy and Nahrawy 2021). Cyanobacteria produce siderophores in a wide variety of environments (Deng 2021; Årstøl and Hohmann-Marriott 2019).

**Fig. 1:**
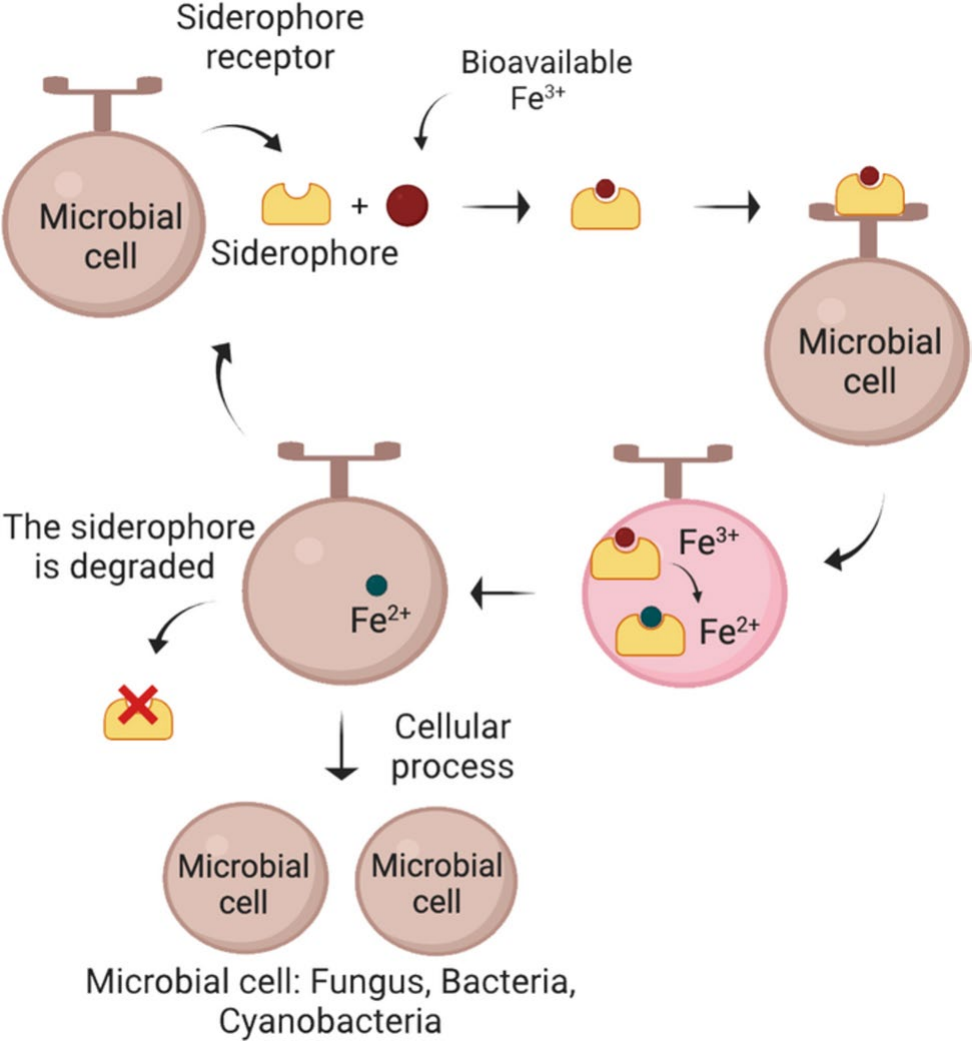
A schematic representation of the mediated iron uptake by microbial siderophores is shown. Siderophores are secreted by microbial cells under iron-limited conditions. They form a complex with bioavailable ferric ions (Fe^3+^) and then bind to a specific receptor on the cellular outer membrane to transport the complex into the cell. Inside the cytosol, the ferric ion (Fe^3+^) is reduced to a ferrous form (Fe^2+^) that dissociates from the siderophore because it has less affinity. The siderophore can be reused or degraded in the cytosol by hydrolysis. Ferrous ions are used by microbial cells in different cellular processes such as protein function, synthesis of amino acids, DNA metabolism, growth, respiration, and photosynthesis

In bacteria, siderophores bind firmly to iron (Fe^+3^) for their transport into the cell using specific siderophore receptors (Fig. 1). Periplasmic siderophore-binding proteins and ATP-dependent transporters in Gram-positives are involved in the transport of siderophore-Fe^+3^ complexes (Roskova et al. 2022).

Regarding Gram-negative bacteria, the transport of siderophore-Fe^+3^ involves an outer membrane receptor, a periplasmic binding protein, and a cytoplasmic membrane ATP-binding cassette (ABC) transport system (Roskova et al. 2022). Although their primary function is to provide iron to microorganisms for growth, there are several applications in areas including ecology, agriculture, bioremediation, biosensors, and medicine (Khan et al. 2018; Khasheii et al. 2021a). In the healthcare field, we are facing a constant increase of multidrug-resistant microorganisms (Tonziello et al. 2019). Besides, a lack or ineffective treatments and vaccines for some diseases like malaria and cancer turned them into public health problems nowadays (Khasheii et al. 2021a). This situation makes looking for new strategies to solve these problems necessary. Siderophores’ properties offer a viable alternative to face these clinical problems, and a good number of publications dealing with these aspects are continuously published. Our research group uses genetic-mining techniques to search for potential new siderophores. Using *Embleya* sp. as a working model, we search for novel siderophores with potential use in medicine and other fields (Caicedo-Montoya et al. 2021; Gómez-Román 2021). In this review, we aimed to integrate the microbial siderophore’s current advances, opportunities, and challenges as viable therapies to fight against diseases that represent crucial public health problems in medicine.

## Siderophores used as antimicrobial agents

One of the most critical global concerns about human health is the emergence of multi-resistant drug bacteria and, consequently, the aggravation of nosocomial infections (Sousa et al. 2021). For this reason, the World Health Organization (WHO) in the year 2017 published a list of the highest-priority bacteria requiring new antibiotics, firstly including Gram-negative pathogens like *P. aeruginosa*, *Acinetobacter baumanni*, and *Enterobacteriaceae*. In second place are pathogens like *Staphylococcus aureus* and *Streptococcus pneumoniae*, among others (WHO 2017).

Siderophores have been the focus of attention as an alternative for promoting and enhancing the absorption of antibiotics. In the next paragraphs, we are presenting new examples of siderophores with antimicrobial activity.

Cefiderocol, is a synthetic compound made from cephalosporin and a catechol-type siderophore with significant antimicrobial activity against clinical isolates of *Enterobacteriaceae* worldwide, which frequently exhibit resistance to third-generation cephalosporins (Syed 2021). This conjugate was first identified in the 1990s; however, the clinical stage did not progress due to their poor physicochemical properties, and since then, the research was focused on the structure and activity of the compound, developing several analogous modified at C-3 and C-7 side chains culminating in the cefiderocol (Sato et al. 2019). On November 14, 2019, this was the first siderophore cephalosporin approved for human treatment by the Food and Drug Administration (FDA) under the brand name Fetroja® (Syed 2021). The cefiderocol utilizes the iron transport system by binding the catechol moiety with extracellular iron, which results in potent antibacterial activity (Zhanel et al. 2019). Evidence of the effectiveness of this compound has been demonstrated in vitro against clinically relevant bacteria isolates expressing unrelated so-called minor carbapenemases. Here, it was observed that cefiderocol, along with ceftazimide-avibactam and meropenem-vaborbactam, are effective against severe, uncommon infections caused by microorganisms producing carbapenemases (Sadek et al. 2023). In addition to this, the in vitro evaluation of cefiderocol against *Enterobacterales*, *Acinetobacter baumannii*, and *Pseudomonas aeruginosa*, among others, showed that this compound was able to inhibit 94% of the isolates (Padovani et al. 2023), and in a surveillance study conducted with Gram-negative bacterial clinical isolates from North America and Europe (2014 to 2019) showed that cefiderocol inhibits in average > 90% of *Enterobacterales*, > 96% of *A. baumannii* and > 99% of *P. aeruginosa* isolates non-susceptible to diverse carbapenems (Karlowsky et al. 2022). These results are also promising in clinical trials where cefiderocol has been successfully used to treat severe infections with carbapenem-resistant Gram-negative bacteria, achieving clinical and microbiological cure of up to 84.6% of the patients 7 days after the treatment has ended (de la Fuente et al. 2023). It is worth mentioning that during the last years, bacteria with low susceptibility or even resistance to cefiderocol have been detected (Karakonstantis et al. 2022; Moon and Huang 2023; Padovani et al. 2023), which makes it necessary to continue searching for new siderophore-antibiotic hybrid conjugates for treating bacterial infections.

Searching for novel drugs sometimes leads back to compounds known for decades. An example of this occurred with albomycins, discovered in the 1940s and produced by *Streptomyces griseus* (Rodrigues et al. 2021). Three of these natural compounds have been synthesized (δ_1_ (1a), δ_2_ (1b), and ε (1c)), and their effect against infections by microorganisms such as *Streptococcus pneumoniae* and *Staphylococcus aureus* leads to the promise of candidates for treatments (Al Shaer et al. 2020). They are composed of a tri-d-*N*-hydroxy-L-ornithine peptide siderophore and a thionucleoside warhead with six consecutive chiral centers differing only in the pyrimidine nucleobase’s C4 substituent (Rodrigues et al. 2021). It has been observed that the albomycin δ_2_ is more efficient than the other albomycins, as well as other antibiotics such as ciprofloxacin, vancomycin, and penicillin (Lin et al. 2018). In the last decade, efforts have been made to understand the structure, mechanism of action, biosynthesis, immunity, and chemical synthesis of analogs to design novel antimicrobial compounds (Wang et al. 2022).

Another exciting compound is anachelin, a cyanobacterial siderophore produced by combining non-ribosomal peptide synthase (NRPS) systems with polyketide synthase (PKS). This siderophore can bind strongly with metal-oxide surfaces and hybridize with vancomycin through a polyethylene glycol (PEG-3000) linker (Arstol and Hohmann-Marriott 2019). This study demonstrated that anachelin could firmly attach the complex to TiO2 surfaces and that PEG suppresses the attachment of bacteria to dead cells or cell material. Vancomycin maintains its biological activity against *B. subtilis* in this complex and shows good stability (Table 1).

**Table 1:** The potential of siderophores for different medical applications

Medical application	Effect	Siderophore- or hybrid-conjugated	Target	References
Antimicrobial	Antibiotic against carbapenem-resistant bacteria	Cefiderocol	*Pseudomonas* spp., *Acinetobacter* spp., *Stenotrophomonas* spp., *Burkholderia* spp.	Syed (2021)
	Antibiotic against Gram-positive bacteria	Albomycins	*Streptococcus pneumoniae, Staphylococcus aureus*	Rodrigues et al. (2021)
		Anachelin-vancomycin	*Bacillus subtilis*	Årstøl and Hohmann-Marriott (2019)
	Trojan horse	Enterobactin analog-ciprofloxacin derivative	*P. aeruginosa*, *B. pseudomallei*	Loupias et al. (2023)
		Enterobactin analog-trimethoprim	*E. coli*	Kim et al. (2023)
Antimalarial	Inhibitor of essential metabolism	Desferrioxamine B	*Plasmodium falciparum*	Rehan et al. (2022)
Anticancer	Reduction in proliferation and tumor growth	Desferrioxamine B	RAW 264.7 (murine macrophage) MCF-7 (human breast) K 562 (human leukemia)	Gokarn et al. (2017)
		Exochelin-MS	RAW 264.7 (murine macrophage)	
		Mycobactin	HEPG2 (human liver)	
Vaccines	Immune stimulation	Enterobactin and salmochelin conjugated to cholera toxin subunit B	Adherent-invasive* E. coli*	Gerner et al. (2022)
		Yersiniabactin-cBSA Aerobactin-cBSA	Uropathogenic* E. coli*	Pecoraro et al. (2022)
		Klh-Enterobactin	Avian pathogenic *E. coli*	Wang et al. (2023)

## Antimalarial applications of siderophores

The *Plasmodium falciparum* parasite, a drug-resistance problem of global interest, highly depends on a sufficient supply of iron for infection (Maya-Maldonado et al. 2021). For this reason, efforts have been focused on an approach that seeks to deprive the parasite of iron as a control measure for malaria (Ganley et al. 2020). It has been demonstrated that desferrioxamine B (DFO-B), synthesized by *Streptomyces* sp. (Rehan et al. 2022) and commercialized as Desferal®, can be used successfully against *P. falciparum.* Thus*,* acting as an iron chelator inhibits the essential metabolic processes of the parasite. Also, the synthesis of natural and artificial iron chelators and their conjugation with peroxide-based antimalarials like ozonides has proved an excellent approach to this purpose (Tiwari et al. 2023). In 2020, there were an estimated 241 million cases of malaria worldwide, and the estimated number of deaths stood at 627,000 (WHO 2022). Because the fight against this disease is focused on controlling the *Anopheles* mosquito that transmits the parasite (Sougoufara et al. 2020) and on vaccination, many of these works have not advanced. Nevertheless, the recent approaches in chemoprevention (Greenwood et al. 2021) make necessary the development of new alternatives in malaria treatment, so these strategies should be soon resumed.

## Siderophores used as anticancer agents

Iron is a vital nutrient for the cells; however, when we talk about cancer, the requirements of this element in malignant cells are significantly higher due to its replication rate (Guo et al. 2021). Thus, decreasing iron concentrations in the cellular environment with siderophores has reduced proliferation and tumor growth (Pita-Grisanti et al. 2022). It has been also reported the effect of the bacterial siderophores exochelin-MS and mycobactim, produced by *Mycobacterium smegmatis*, and desferrioxamine B (DFO) obtained from *S*. *pilosus*. MTT cell proliferation assay determined that these compounds caused a significant decrease in the replication of malignant human cell lines from the breast, liver, and leukemia in concentrations that did not affect the growth of normal cells after 18–24 h of exposure (Gokarn et al. 2017). DFO has been demonstrated to have antitumor activity in clinical trials, but a plasma half-life of 20 min approximately limits the therapeutic potential; in this regard, efforts have been made to increase the pharmacokinetics by conjugating DFO with biocompatible polymers as poly(ethylene glycol)‐poly(aspartic acid) observing an antiproliferative effect in a model of subcutaneous tumor (Komoto et al. 2021). Another approach of combinatorial therapy has been explored; here, the use of DFO with α-cyano-4-hydroxy cinnamate (suppressor of lactate excretion) resulted in an efficient anticancer therapy where the increased lactate levels in HeLa cells triggered by DFO served as specific target preventing its excretion resulting in high acidity and damage to the cell (Fujisawa et al. 2022).

The effect of the bacterial catecholate-type siderophore enterobactin as a potential anticancer agent has also been evaluated (Saha et al. 2019). The authors demonstrated a cytotoxic effect of enterobactin on two monocyte tumor cells but not on bone marrow–derived macrophages. Among the observed effects in this experiment, both cell lines showed a large intracellular labile iron pool, and the siderophore altered their homeostasis. Secondly, enterobactin disrupted the reactive oxygen species (ROS) generation by the mitochondria in a dose-dependent manner. Together, these two properties can cause apoptosis of cancer cells (Saha et al. 2019).

Nosrati et al. 2021 designed a theragnostic (from therapeutics and diagnostics) platform based on superparamagnetic iron oxide nanoparticles-pyoverdine (SPION/PVD) conjugate bound to a MUC1 aptamer (MUC1Apt) loaded with the anticancer drug doxorubicin. In this study, by the characterization and evaluation of this conjugate in vitro*,* ex vivo, and in vivo, they concluded that the formulation SPION/PVD/MUC1APT/DOX provides an efficient dual modality–targeted nanoparticle that could be employed for clinical cancer diagnosis and therapy.

With these results, we can strongly propose to extend the study on using siderophores as adjuvants in cancer treatments.

## The use of siderophores as vaccines

Siderophores can act as haptens that can elicit an immune response when conjugated to a carrier protein. For example, immunization with enterobactin or salmochelin conjugated to cholera toxin subunit B reduced intestinal colonization and severity of adherent-invasive *E. coli* colitis in a mouse model (Gerner et al. 2022). Some other examples are the conjugated siderophores yersiniabactin (Ybt) or aerobactin (Aer) with a cationic form of bovine serum albumin (cBSA). Their use resulted in an adaptive immune response by targeting bacterial stealth siderophores and protecting against urinary tract infections (UTIs) caused by uropathogenic *E. coli* when induced in mice immunized with cBSA-Aer or cBSA-Ybt. In addition, an OVA-VIB conjugate capable of eliciting a selective antigenic response in mice was obtained by covalently linking the vibriobactin (VIB) analog to the carrier proteins ovalbumin (OVA) (Pecoraro et al. 2022). Wang et al. (2023) performed a chicken challenge assay to evaluate the immunogenicity and protective efficacy of a keyhole limpet hemocyanin-Ent conjugate vaccine against avian pathogenic *E. coli* infection in chickens. This vaccine elicited a strong systemic immune response and high serum and gut-specific IgY titers in vaccinated chickens. Enterobactin conjugate vaccination controls systemic and enteric infections of bacterial pathogens that depend on this siderophore for iron acquisition, such as *Campylobacter jejuni* (Cui et al. 2020).

Progress was made in using mutant TonB strains and subunits of the protein as vaccine candidates against several microorganisms, including *A. baumannii*, *Salmonella enterica*, and *P. aeruginosa*, using the approach of targeting the transporters for siderophore-iron complexes (Wang et al. 2021).

## Siderophores used as drug-delivery compounds

Siderophores have been the focus of attention as an alternative for promoting and enhancing the absorption of antibiotics in use by a mechanism named Trojan horse. This mechanism consists of siderophores conjugated to a drug (such as an antibiotic) that facilitates uptake into pathogenic cells. When the drug is unable to cross the bacterial membrane, it can bind to a siderophore. The generated drug-Fe^+3^ siderophore is recognized by its corresponding receptor used to transport iron and, thus, shuttle the drug into the cell. In this way, the administered antibiotic can kill pathogens, either through the antibacterial activity of the compound itself or by preventing the formation of the iron-siderophore conjugate (Khasheii et al. 2021b; Peukert et al. 2023; Nazli et al. 2022). Studies on *P. aeruginosa* supported that a catechol-type siderophore-antibiotic conjugate is a promising tool for treating infections. Proteomics and RT-qPCR assays showed that the conjugate promotes the expression of its iron uptake transporter in *P. aeruginosa*, making it a promising vector for antibiotic delivery (Perraud et al. 2020). Knowledge of the receptor and transporter proteins is essential for this approach, as artificial siderophores can also be generated. Such is the case of the membrane protein TonB from *P. aeruginosa*, a nutrient transporter that also allows the internalization of iron-siderophore complexes. Loupias et al. (2023) describe the synthesis of a conjugate between a ciprofloxacin derivative and a synthetic catechol-based siderophore. The conjugate was four times more potent against *P. aeruginosa* and *Burkholderia pseudomallei* than ciprofloxacin alone. On the other hand, a conjugate was prepared between a library of catechol-type siderophore analogs and the antibiotic trimethoprim, which showed activity only against *E. coli* (Kim et al. 2023).

Interestingly, the TonB-dependent transporter FoxA of *P. aeruginosa* transports not only the siderophores ferrioxamine B, nocardamine, and with lower affinity bisucaberin but also the peptide antibiotic thiocillin (Chan et al. 2023). The same applies to the FpvB transporter, which transports the antibiotic thiostrepton and the siderophores pyoverdin, ferrichrome, and ferrioxamine B (Chan and Burrows 2023). This condition is relevant to drug development because the more significant the induction of both siderophore transporters is, the greater the antibiotic transport.

## Visualizing siderophores as targets

In the search for novel alternatives, researchers have focused on targeting virulence factors to avoid the selective pressure for resistance (El-Aleam et al. 2021). Since iron acquisition by bacterial siderophores is considered a virulence factor, one approach is targeting potential inhibition sites like siderophore biosynthesis, outer membrane receptors, iron release enzymes, and signal receptors (Post et al. 2019).

For example, baulamycin A and B from *Streptomyces tempisquensis* are two molecules that inhibit the synthesis of the siderophores staphyloferrin B from *Staphylococcus aureus* and petrobactin from *Bacillus anthracis*. They can penetrate bacterial barriers and inhibit the growth of both Gram-positive and Gram-negative species (Ribeiro and Simões 2019). Peukert et al. (2023) produced conjugates between synthetic siderophore analogs of enterobactin with selected peptides from the TonB box. The accumulation of TonB peptides inhibits iron transport, decreasing the growth of the pathogenic bacterium *P. aeruginosa*.

The modification of natural products for the targeting of pathogen siderophores is a valuable strategy. This is the case of human siderocalin (lipocalin 2), a protein capable of sequestering enterobactin and bacillibactin from *E. coli*. Siderocalin has been engineered in the ligand pocket formed by the loops connecting its classical β-barrel structure, resulting in a protein that binds to petrobactin from *B. anthracis*, suppressing its growth (Dauner and Skerra 2020).

## Siderophores used as biosensors for medical diagnosis

Due to their simplicity, high sensitivity, and potential capability for real-time and in situ analysis, biosensors have been used in various applications in advanced medical diagnostics, environmental monitoring, genomics, nanobiotechnology, food processing, and agricultural industries, among others (Ramya et al. 2022; Ribeiro et al. 2022). Siderophore-based biosensors have provided new ways of detecting metal ions such as calcium, aluminum, zinc, and molybdate (Singh et al. 2020). The development of new nanoscale materials and technologies has enabled powerful analytical platforms to detect low concentrations of metal ions and numerous pathogens (Nosrati et al. 2018; Hu et al. 2019; Li et al. 2021). The microbial pathogen-detection techniques rely on the siderophore immobilization on a monolayer gold-coated surface, and their high affinity and specificity to the bacterial cell-surface receptors. Bacterial attachment can be detected by Fourier transform infrared spectroscopy (FTIR) and fluorescence microscopy (Bhadra et al. 2018). Thus, siderophores specificity can be used for species-specific bacterial identification (Zheng and Nolan 2012).

The major challenge in designing detection methods and tools is identifying suitable siderophores. Although some siderophores have the potential to be used as biosensors, most of them still need to be characterized.

For greater precision, siderophore-based detection can be combined with different analytical techniques, such as high-performance liquid chromatography, gas chromatography, and enzyme-linked immunoassay. The use of aptamers has increased to design siderophore-based biosensors due to their ability to perform accurate and reproducible target detection (Danesh et al. 2018) (Table 2). The advantages of using DNA aptamers are the reproducibility of their production (chemical synthesis), showing less steric hindrance than other molecules, being very stable at room temperature, and can bind very specifically to their ligand (Singh et al. 2020).

**Table 2:** Examples of siderophores with biosensor applications

Siderophore	Biosensor composition	Use	References
Catechol	Gold particles are functionalized with catechol and covered with *N*-hydroxyethyl acrylamide	Iron detection	Phillips et al. (2015)
	Nanoparticles of Fe_3_O_4_ are functionalized with the siderophore 2,3-dihydroxybenzoylglycine	Aluminum detection	Raju et al. (2017)
Biscatecholate–monohydroxamate siderophore	A biscatecholate–monohydroxamate siderophore linked to a biotin through three polyethylene glycols is integrated into a localized surface plasmon reverberation (LSPR) sensor	*Acinetobacter baumannii* detection	Hu et al. (2019)
Desferrioxamine	The biosensor is based on three modules, one of which is a copolymer of chloroprotoporphyrin IX Fe^3+^-polyethylene glycol-desferrioxamine	*Helicobacter pylori* detection	Wang et al. (2021)

Regarding diagnoses, siderophores have also been used directly to detect fungal infections. An example is the invasive fungal pulmonary aspergillosis infection caused by *Aspergillus fumigatus,* recently designed as one of the four critical priority fungal pathogens by the World Health Organization (https://www.who.int/publications/i/item/9789240060241). This fungus is a life-threatening disease, causing more than 1 million deaths yearly in immunocompromised hosts from the more than 14.5 million reported cases (Škríba et al. 2018). The infection can be diagnosed in humans with very high confidence (92%) in just a few hours, just determining the triacetylfusarinine C (TafC) concentration, a siderophore produced by *Aspergillus fumigatus* (Dobiáš et al. 2022). A challenge in these applications is developing simple, precise, reproducible, rapid, and cost-effective diagnostic methods for detecting pathogens using urine or other accessible biological samples.

## Concluding remarks and future prospects

Siderophore inquiries are a field of incredible scientific intrigue. Their significance in nature is evident as they assume an essential role in the metabolism of several microorganisms. However, a better understanding of the compound structures of different siderophores will assist with comprehending their physiological capacity and give rise to other inventive applications. Regarding the biomedical or pharmaceutical fields, besides their role in drug delivery, antimicrobial, antimalarial, and anticancer drugs, as well as vaccines, siderophores have other prospects. These include their use in diagnosing cancer and fungal diseases, treating iron and aluminum overload disorders (for example, sickle cell anemia), and removing transuranic elements from the body. Siderophores can also act as anti-arteriosclerotic agents, as it has been proven that lipid degradation depends on the degree of iron accumulation in arteriosclerotic lesions. They can also influence interactions between bacteria and between bacteria and hosts, which may have implications for infection control and microbiome modulation. Thus, the future looks bright for siderophores applications in medicine. We expect active incorporation and participation of the pharma industry in these developments as this is crucial to crystallize some of the siderophore’s biomedical or pharmaceutical possibilities. This action will also ensure faster incorporation of the siderophore’s abilities into the medical practice.

Besides the medical field, there is a high and diverse demand for siderophores in other areas, including ecology, agriculture, and bioremediation. To feed such a demand, the extremophiles and mangrove ecosystems are a vast source to look for novel microbial siderophores. Exploiting their benefits for all living beings and the environment is necessary. Consequently, it is essential to identify and characterize novel microbial siderophores from those habitats to identify new biotechnological applications and fine-tune the current ones.
